# Supplementary material for: Improved mammalian retromer cryo-EM structures reveal a new assembly interface
Source: J Biol Chem. 2022 Sep 26;298(11):102523. doi: 10.1016/j.jbc.2022.102523 (PMC9636581; doi:10.1016/j.jbc.2022.102523)
Supplement: Supplemental Figure Legends [file mmc1.docx]

**Supplemental Figure Legends.**

**Figure S1. CryoEM image and data processing work flow for updated retromer heterotrimer and VPS35/VPS35 sub-structure.** Particles were auto-picked from three combined datasets using a 3D starting model (details in Methods). Particles were separated into 2D classes based on biochemical species (heterotrimer, flat chains). The left branch shows data processing pipeline for heterotrimers, and the right branch shows processing for the VPS35/VPS35 sub-structure reconstructed from flat chains. 3D reconstructions were generated for each species. Fourier Shell Correlation (FSC) plots showing masked and unmasked resolution estimates from RELION are shown for each structure or sub-structure; the grey line marks the 0.143 cut-off. All scale bars represent 10 nm.

**Figure S2. CryoEM image and data processing work flow for retromer dimers and sub-structure.** Particles were auto-picked from a published dataset (Kendall, *Structure* 2020) using a 3D starting model generated from EMD-21117. Dimer particles were separated from other species during 2D classification, and an *ab initio* model was generated from the particle stack. This model was used as a reference model for 3D reconstructions generated for both the dimer (left branch) and the VPS35/VPS35 sub-structure (right branch). Fourier Shell Correlation (FSC) plots showing masked and unmasked resolution estimates from RELION are shown for each structure or sub-structure; the grey line marks the 0.143 cut-off. All scale bars represent 10 nm.

**Figure S3. Analysis of retromer dimer map handedness.** Map handedness was systematically analyzed by performing random rigid-body fits of real-space refined retromer models into each map (details in Methods). (A) Fitting histogram for original handedness map showing counts of random rigid-body fits and cross-correlation values (Chimera). Magenta star marks top fit. (B,C) Two views of top fitted model in Coulomb potential map. Maps were generated using Chimera and are shown at 7σ contour level; VPS35 in red ribbons; VPS26 in blue ribbons; VPS29 in green ribbons. (D) Fitting histogram for flipped map handedness showing counts of random rigid-body fits and cross-correlation values (Chimera). (E, F) Two views of top fitted model in Coulomb potential map. Maps were generated using Chimera and are shown at 7σ contour level. This analysis suggests the original handedness represents the correct handedness.

**Figure S4. CryoEM image and data processing work flow for retromer 3KE mutant.** Particles were auto-picked from a single dataset using an initial 3D starting model generated in preliminary processing (top box; further details in Methods). 3KE mutant particles were separated from other biochemical species during 2D classification, and an *ab initio* model was generated from the particle stack. This was used as a reference model for 3D reconstructions generated for both the 3KE particle (left branch) and its sub-structure focusing on the chain link (right branch). Fourier Shell Correlation (FSC) plots showing masked and unmasked resolution estimates from RELION are shown for each structure or sub-structure; the grey line marks the 0.143 cut-off. All scale bars represent 10 nm.

**Figure S5. Analysis of retromer 3KE mutant map handedness.** Map handedness was systematically analyzed by performing random rigid-body fits of real-space refined retromer models into each map (details in Methods); cross correlation coefficients and number of hits for each fit are shown. (A) Fitting histogram for original map handedness map showing counts of random rigid-body fits and cross-correlation values (Chimera). Magenta star marks top fit. (B, C) View of top fitted model in Coulomb potential map. Maps were generated using Chimera and are shown at 7σ contour level; VPS35 in red ribbons; VPS26 in blue ribbons; VPS29 in green ribbons. (D) Fitting histogram for original map handedness showing counts of random rigid-body fits and cross-correlation values (Chimera). (E, F) View of top fitted model in Coulomb potential map. Maps were generated using Chimera and are shown at 7σ contour level. This analysis suggests the original handedness represents the correct handedness.

**Figure S6. Reconstruction of the 3KE particle sub-structure.** (A, B) Reconstructions of retromer 3KE sub-structure particle (A) and a close-up view (B) centered on the “chain link” mediated by VPS26 and VPS35. Coulomb potential maps generated using CCP4MG are shown at 6σ contour level. (C) Local resolution and angular distribution (D) of 3KE sub-structure particle.

**Figure S7. Analysis of retromer interfaces.** (A, B) Equivalent views of published retromer arches are shown with their respective Coulomb potential maps from EMDB. (C) While individual retromer heterotrimers can be fitted as rigid bodies into the dimer map (cf. Figure 2; Figure S2), assembled arches are very poorly fitted as a single rigid body into the dimer map (rigid body fits in Chimera; full details in Discussion). (D, E) Two views rotated by 45 degrees of the N-VPS26A/N-VPS35 interface observed between a N-VPS35 (red ribbons) and N-VPS26A (blue ribbons) in a neighboring molecule; transparent electrostatic surface views are overlaid to demonstrate surfaces. The first helix of VPS35 (residues 12-36; labelled “N”) interacts primarily with two b-strands (residues 48-56; 105-111) and two loops (residues 56-63; 101-105) in N-VPS26A. The overall shape of each subunit is complementary to its binding partner. (F) VPS26 conservation (ConSurf) mapped onto the VPS26A structure. The N-terminal interface with VPS35 does not exhibit high conservation levels across eukaryotes, suggesting this interface may not occur in all organisms with retromer.
